# USING PARADATA AND MANUAL REVIEW TO DETECT AUTOMATED ONLINE RESEARCH SURVEY PARTICIPATION

**DOI:** 10.1093/geroni/igad104.1176

**Published:** 2023-12-21

**Authors:** Jeffrey Boon, Cathy Maxwell

**Affiliations:** Vanderbilt University Medical Center, Nashville, Tennessee, United States; Vanderbilt University, Nashville, Tennessee, United States

## Abstract

With an increase in health research recruitment on social media, there is also an increase in prevalence of software known as bots auto-enrolling in online studies. Despite efforts to prevent automated fraudulent enrollment, bots have become more sophisticated with some able to bypass a CAPTCHA and generate false email addresses. This session describes such fraudulent enrollment in one study and our efforts to detect it. For an online study of family caregivers of people with dementia, we recruited via family caregiver groups on Facebook. Potential participants could proceed to the online study and immediately answer screening questions and complete the study. A CAPTCHA was used to screen non-human respondents, but we detected rapid enrollment compared with other methods, raising suspicions. Paradata generated in the process of survey administration and unusual email addresses flagged suspected bot responses. To screen the responses, we reviewed the time to complete surveys compared with known human responses. We also noted email addresses that consisted of long alphanumeric usernames not common with most human respondents. After screening for suspicious respondents, we attempted to contact the email addresses of borderline cases to verify they were real respondents. Only one of 157 (0.006%) were determined to be a real respondent. Lessons learned from this experience include using paradata as part of data quality monitoring, using survey design methods to elude automated bots, and the value of researchers interacting with participants during enrollment.

